# OpticalTrust: A Sensor-to-Blockchain Framework Using Free-Space Optical Communication

**DOI:** 10.3390/s24237797

**Published:** 2024-12-05

**Authors:** Parveen Bajaj, Aman Kataria, Vikram Puri, Sachin Gupta, Hong Min

**Affiliations:** 1 University Institute of Engineering, Chandigarh University, Gharuan, Mohali 140413, India; erparveen@rediffmail.com; 2 University Centre for Research and Development, Chandigarh University, Gharuan, Mohali 140413, India; 3 School of Computer Science, Duy Tan University, Da Nang 550000, Vietnam; purivikram@duytan.edu.vn; 4 Institute of Research and Development, Duy Tan University, Da Nang 550000, Vietnam; 5 Department of Robotics and Control, School of Electronics and Electrical Engineering, Lovely Professional University, Phagwara 144411, India; sachin.23305@lpu.co.in; 6 School of Computing, Gachon University, 1342 Seongnamdaero, Sujeong-gu, Seongnam-si 13120, Republic of Korea

**Keywords:** sensor network, free-space optical communication, BER, OptiSystem, EDFA, blockchain, interplanetary file system (IPFS), smart contract

## Abstract

In the dynamic landscape of the tech industry, the escalating requirement for swift and secure data transmission has catalyzed innovation in integrated communication systems. Free-Space Optics (FSOs) has emerged as a promising contender in optical communications. While conventional optical fiber systems can achieve bit rates of up to 40 Gbps with proper design, they are limited primarily by electronics rather than semiconductor laser capabilities. This study presents an integrated framework that combines FSOs, blockchain technology, and sensor networks to address challenges in data transmission, security, and environmental adaptation. This study analyzes FSOs system performance through the Quality (Q) Factor and Bit Error Rate (BER), comparing systems with and without Erbium-Doped Fiber Amplifiers (EDFAs) across various bit rates (8, 12, 16, and 20 Gbps) and transmission distances (5–25 km). To enhance data security and reliability, a blockchain architecture is incorporated with smart contracts and an InterPlanetary File System (IPFS) for storing and validating results generated from FSOs simulation. Additionally, this study explores the design of sensor network models for FSOs technology by investigating how distributed sensor arrays can be theoretically integrated with FSOs systems, with testing focused on FSOs performance and blockchain implementation.

## 1. Introduction

The dynamic field of telecommunications continues to undergo transformative changes, particularly in data transmission technologies. Traditional residential and commercial networks relied heavily on copper-based infrastructure for phone and internet services [1]. This push for innovation aims to overcome the constraints of traditional copper infrastructure and meet the growing demand for amplified data quantity transmission rates and enhanced network performance. Among these, wireless transmission technologies—including both radio frequency (RF) and FSOs—have proven to be strong options [2,3].

FSOs uses open space to transmit light beams, which creates a channel for data interchange [4]. Modern FSOs systems aim to achieve high transmission rates, while conventional optical fiber systems can already operate at bit rates of up to 40 Gbps with proper design, primarily limited by electronics rather than the optical technology itself [5]. FSOs demonstrates superior agility in establishing connections for both unicast and multicast configurations, outpacing legacy technologies such as Radio over Fiber (ROF) and traditional optical fiber systems [6]. FSOs systems achieve full-duplex communication through indoor-deployed optical transceivers with integrated laser transmitters and receivers, offering the dual advantages of eliminating RF radiation risks and avoiding extensive underground cabling while maintaining optimal resource utilization through its high-capacity spectrum [3,7,8].

Simultaneously, with these advancements, sensor networks have become a crucial element in the changing paradigm of data transmission technologies. These networks comprise globally dispersed independent sensors that observe physical or environmental variables and collaboratively transmit their data over the network to a central hub [9]. Sensor networks can be seamlessly integrated with FSOs and other data transfer technologies to create robust, intelligent systems for data collection and transmission. For instance, in smart city applications, sensor networks can gather real-time data on traffic patterns, air quality, and energy usage, which can then be rapidly transmitted via FSOs links to central processing units for analysis and decision-making [10]. This synergy between sensor networks and advanced data transfer technologies like FSOs is paving the way for more efficient, responsive, and interconnected systems across various domains, from environmental monitoring to industrial automation [11]. In addition to these advancements in data transfer and sensor technologies, blockchain has emerged as a transformative force in securing and managing data across networks. Blockchain technology, with its decentralized and immutable ledger system, offers a novel approach to enhancing the security, transparency, and efficiency of data management in FSOs and sensor network systems.

Images obtained by optical spectrum analyzers are crucial for guaranteeing the dependability, efficiency, and sustained prosperity of FSOs systems [12,13]. Engineers and researchers can use these images and the information they provide to improve FSOs communication, making it faster and more secure for different uses. Storing this image securely is crucial due to potential problems that could compromise data integrity, data accessibility, and data provenance. To address these challenges, blockchain technology is the appropriate option. The solutions are as follows:

1. Immutable: Blockchain’s cryptographic nature ensures that any data within the network is tamper-proof. Every single image on the network is hashed and preserved.
2. Secure Access and Authentication: Blockchain facilitates the implementation of safe access control mechanisms via private and public key cryptography. Stored photos can be accessed or denied access based on predetermined rules enforced through smart contracts. This guarantees that only authorized entities may securely access and distribute the images.
3. Transparency: Blockchain preserves an unchangeable and time-ordered history of all transactions connected to images. Every transaction, including the addition, retrieval, or modification of an image, is documented on the blockchain, ensuring a clear and verifiable path that simplifies the process of monitoring transaction records.

This study presents several noteworthy contributions to the field. The key findings and innovations of our research are as follows:

- In this study, a comparative analysis is proposed for systems with and without EDFAs.
- This study also evaluates the FSOs model using Q-Factor and BER (Bit Error Rate) measurements.
- This study focuses on the integration of sensor networks, FSOs, and blockchain technology.
- To evaluate the blockchain network, three scenarios are considered: default, optimized, and congested network conditions.
- The evaluation is based on parameters, including throughput, time, and bandwidth.

## 2. Related Work

This section discusses the related work on the integration of sensor networks with blockchain technology and FSOs.

### 2.1. Sensor Network and Blockchain

In recent years, the synergy of blockchain technology with sensor networks has garnered significant attention due to its capabilities to enhance data security, integrity and transparency in numerous applications. Recent research has directed its attention on harnessing the intrinsic characteristics of blockchain technology to enhance data management in sensor networks. In [14], the authors propose a novel approach to enhancing data security in wireless sensor networks (WSNs) by integrating blockchain technology with data transfer protocols. The system employs small-area WSNs, each with a primary “mobile database” node utilizing embedded microcontrollers, to create a robust IoT-based network architecture. By adapting blockchain principles to sensor data records and implementing private cloud-end functionality, the research aims to significantly improve data reliability and tamper-resistance in WSN structures. A decentralized system is proposed in [15] to enhance the security, privacy, reliability and autonomy of IoT ecosystems. By integrating blockchain technology and cryptographic tools, the purpose of this study is to protect data integrity and availability. Awan [16] proposped an encryption and trust evaluation model based on blockchain technology for wireless sensor networks. The model authenticates aggregator nodes and sensor nodes using public and private blockchains, respectively, and employs trust value computation to identify and eliminate malicious nodes. To secure the sensor node data, RSA encryption is applied. There are some studies focused on WSN protocols using blockchain technology. A systematic review of the integration of blockchain technology (BCT) and the Internet of Things (IoT) is presented in [17] to address security and privacy challenges. The study examines the basic principles, architectures, protocols, and consensus algorithms of both BCT and IoT, as well as the challenges of their integration. It analyzes various approaches to leveraging blockchain’s security features in IoT ecosystems, categorizing applications based on characteristics such as development level, blockchain type, and consensus algorithm. In [18], the authors propose a novel blockchain-based authentication protocol for WSNs to address security vulnerabilities in existing protocols, particularly ID spoofing attacks. The research leverages blockchain’s cryptographic security features to enhance data integrity and source verification in WSNs. The protocol incorporates a private blockchain network connecting sensor nodes, cluster nodes, and a base station. The integration approach of blockchain technology and IoT faces a number of challenges, which include the storage capacity and scalability issue, resource constraints of IoT devices, transaction rate limitations, and legal complexities. These challenges stem from the vast amounts of data generated by IoT devices, their limited computational power and energy resources, and the need for high-speed, real-time data processing in many IoT applications. FSOs could potentially address some of these challenges by offering high-bandwidth, energy-efficient, and secure communication links.

### 2.2. Free-Space Optics (FSOs)

Free-Space Optics (FSOs) and fiber optics represent two fundamental approaches in optical communication systems [19]. FSOs technology enables wireless communication through the atmosphere using modulated light beams, capable of achieving multi-gigabit-per-second (Gbps) data rates across urban distances [20,21,22]. In contrast, optical fiber transmission operates by guiding light through a specialized glass structure consisting of a core surrounded by a cladding layer with a lower refractive index, enabling signal propagation through total internal reflection [23,24]. Signal quality in both systems is evaluated through metrics including the Bit Error Rate (BER) and Q Factor, where BER is analyzed using statistical methods like Gaussian and Chi-squared distributions [23], and the Q Factor serves as a critical parameter for assessing transmission pathway performance. These technologies support data transmission rates, with applications ranging from urban communications to island-to-island connectivity [25,26].

Several researchers have made significant contributions to the field of FSOs communication systems and optical amplification strategies. In [27], the authors evaluated the performance of FSOs systems incorporating Erbium-Doped Fiber Amplifiers (EDFAs) under various conditions. The study examined different attenuation levels (0.468 dB/km, 10 dB/km, and 22 dB/km) and assessed system performance through Q-factor and Signal-to-Noise Ratio (SNR) measurements. At a link distance of 1000 m under high atmospheric attenuation, systems with an EDFA achieved a Q-factor of 30.9, compared to 13.3 without an EDFA. Similarly, in [28], the authors conducted a comparative analysis of FSOs systems across three optical transmission windows (850 nm, 1310 nm, and 1550 nm) under different atmospheric conditions, including rain and haze, and examined their effects on FSOs link performance. To improve the performance of FSOs systems in the Middle East region under severe storm conditions, this study [29] proposed a dual-channel FSOs system with EDFA amplification to overcome the limitations of traditional FSOs links. Additionally, the implementation of a 1550 nm wavelength with multiple channels and EDFAs not only achieved superior Bit Error Rate (BER) and Q-factor performance metrics but also extended the effective communication range, presenting a robust solution for FSOs deployment in dust-prone environments. A hybrid FSO/RF system [30] was proposed to demonstrate superior performance, achieving a gain exceeding 1 dB under minimum power conditions, thereby addressing the limitations of traditional FSOs systems during adverse meteorological conditions. The main purpose of this study was to validate that the adaptive joint system, utilizing optimal channel mapping and power distribution algorithms, presents a robust and efficient solution for maintaining reliable broadband connectivity across diverse weather conditions. In [31], the authors provided a comprehensive review of hybrid FSO/RF networks, focusing primarily on practical use cases while highlighting their challenges and implementation issues. The hybrid FSO/RF system demonstrated superior performance over traditional systems by effectively combining the complementary strengths of both transmission methods, resulting in enhanced availability and weather-resistant connectivity. Several studies have focused on applications of hybrid FSO/RF communication systems. For instance, ref. [32] discussed UAV-assisted multi-hop parallel hybrid FSO/RF systems. While most FSOs system research has concentrated on improving signal quality, there is a notable gap in the literature regarding data security, both in experimental settings and real-time applications.

## 3. Methodology

**Figure 1:**
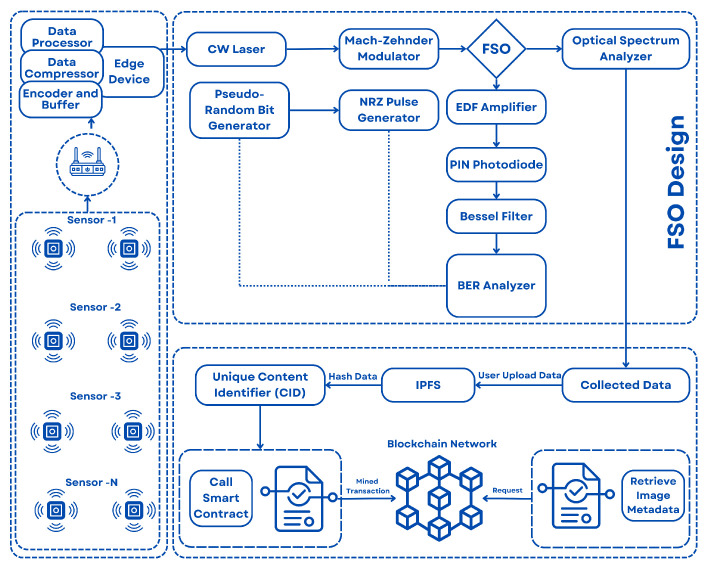
Methodology of the proposed framework.

The proposed approach is categorized into three different sub-sections, such as sensor node, FSOs model design and a decentralized storage system, which are discussed as follows (see Figure 1):

### 3.1. Sensor Nodes

Sensor networks have emerged as a transformative technology in the field of environmental monitoring, industrial automation and smart communication. These distributed systems, comprising numerous spatially dispersed autonomous devices equipped with sensors, have revolutionized data collection and analysis across various domains [33]. The fundamental architecture of a sensor network typically consists of sensor nodes, gateway nodes, and a central server or base station. Sensor nodes, often referred to as “motes”, are compact devices capable of measuring diverse environmental parameters such as temperature, humidity, pressure, or chemical concentrations. These nodes are designed to operate with minimal energy consumption, often relying on battery power or energy harvesting techniques for long-term deployment [34].

Consider a sensor network comprising a set of ν sensors, denoted as $\Omega =\omega_{1},\omega_{2},\ldots ,\omega_{\nu }$. Each sensor $\omega_{i}$ collects data at discrete time intervals τ. The data stream generated by sensor $\omega_{i}$ at time τ can be represented as $\chi_{i}\left(\tau \right)$. The data collection process for each sensor can be modeled as afunction:

(1) $$\chi_{i}\left(\tau \right)=F_{i}\left(\varepsilon \left(\tau \right),\sigma_{i}\right)$$

where $F_{i}$ represents the sensing function of $\omega_{i}$, ε(τ) denotes the environmental state at time τ, and $\sigma_{i}$ represents the inherent noise and inaccuracies of the sensor. These individual data streams are aggregated by a gateway Γ. The aggregation process can be mathematically expressed as:

(2) $$\Psi \left(\tau \right)=\Gamma (\chi_{1}\left(\tau \right),\chi_{2}\left(\tau \right),\ldots ,\chi_{\nu }\left(\tau \right))$$

where Ψ(τ) is the composite data stream produced by the gateway. The edge device E applies a series of transformations to Ψ(τ). These transformations can include filtering, compression, and encoding operations. Let H represent the composite transformation function applied by the edge device. Then:

(3) $$\Psi^{\prime }\left(\tau \right)=H\left(\Psi \left(\tau \right)\right)=H\left(\Gamma \left(\chi_{1}\left(\tau \right),\chi_{2}\left(\tau \right),\ldots ,\chi_{\nu }\left(\tau \right)\right)\right)$$

where $\Psi^{\prime }\left(\tau \right)$ is the processed data stream ready for transmission. The processed data stream $\Psi^{\prime }\left(\tau \right)$ from the edge device is fed into the FSOs system’s data modulator. This process can be represented as follows:

(4) $$M\left(\tau \right)=M(\Psi^{\prime }\left(\tau \right))$$

where M(τ) is the modulated signal, and M is the modulation function. The modulated signal M(τ) is then combined with the continuous wave laser output in the Mach–Zehnder Modulator (MZM) [35]. The optical signal produced by the MZM can be expressed as:

(5) $$S\left(\tau \right)=A_{0}\cos\left(\omega_{0}\tau +\pi M\left(\tau \right)/V_{\pi }\right)$$

where:

- $A_{0}$ is the amplitude of the optical carrier,
- $\omega_{0}$ is the angular frequency of the laser,
- $V_{\pi }$ is the half-wave voltage of the MZM.

This optical signal S(τ) is then amplified and transmitted through free space via the FSOs transmitter optics.

The light signal subsequently undergoes amplification by the EDFA [36]:

(6) $$S^{\prime }\left(\tau \right)=G\cdot S\left(\tau \right)$$

where *G* is the gain of the EDFA. The amplified signal S’(τ) is then transmitted through free space via the FSOs transmitter optics. The received signal R(τ) at the FSOs receiver can be modeled as:

(7) $$R\left(\tau \right)=\eta \cdot S^{\prime }\left(\tau \right)+N\left(\tau \right)$$

where η represents the channel attenuation factor. N(τ) is additive noise in the channel, including thermal noise, and background radiation at the receiver.

### 3.2. FSOs Model Design

This study aims to design and analyze an optical transmission system utilizing both Fiber Optic and Free-Space Optical channels. The analysis will be conducted using OptiSystem, a widely adopted simulation program in optical communications. OptiSystem provides an intuitive interface commonly used in various electrical engineering applications, particularly in optics. The FSOs communication system considered for this study [37] incorporates a CW laser source with a Mach–Zehnder modulator driven by a pseudo-random bit generator through an NRZ pulse generator at the transmitter section, followed by an FSO channel split between an optical spectrum analyzer and EDF amplifier. The receiver section comprises a PIN photodiode and Bessel filter for signal detection and conditioning. The system will be evaluated at data rates of 8, 12, 16, and 20 Gbps over distances ranging from 5 to 25 km, specifically at 5, 10, 15, 20, and 25 km intervals. The design incorporates a Bessel filter and an Erbium-Doped Fiber (EDF) amplifier to enhance signal quality. Performance metrics for the FSOs channel will include Bit Error Rate (BER) and Q-Factor assessments. In this study, the system simulation utilizes a wavelength of 1552.52 nm with an attenuation coefficient of 0.2 dB/km, as specified in Table 1. This attenuation parameter was selected to represent typical FSOs channel losses, including atmospheric absorption and scattering under clear weather conditions, as well as baseline propagation losses in the FSOs channel. Table 1 outlines the key design parameters and their corresponding values chosen for the proposed FSOs communication system simulation in OptiSystem.

### 3.3. Decentralized Storage System

A decentralized storage system is employed for the purpose of storing and overseeing data by utilizing the combined storage capabilities of a network of storage devices rather than relying on a single server or control centre [38,39]. The primary goal of the decentralized storage system is to deliver resilient and secure methods of data storage by harnessing the capabilities of blockchain technology [40]. The primary constituents of the decentralized storage system for this study are:

- Blockchain Network: Blockchain is a decentralized system that combines numerous nodes to collectively maintain a database of transactions occurring within the network [41]. The blockchain network possesses essential qualities such as decentralization, immutability, transparency, and enhanced security in comparison to traditional systems.
- Smart Contract: A self-executing protocol, colloquially termed a “smart contract”, is a digitally encoded covenant inscribed within a distributed ledger infrastructure [42]. It is designed to be self-executing without the involvement of any other entity. The primary objective of smart contracts in decentralized storage is to achieve autonomous execution, ensuring that data remain tamper-proof once deployed on the network, and facilitating secure and transparent transactions [43].
- IPFS: To manage and store a huge amount of data in a decentralized manner, the Interplanetary File System (IPFS) is utilized. IPFS facilitates the storage and sharing of files through a peer-to-peer network [44]. IPFS has been implemented in diverse industries, such as creating offline-native productivity tools, ensuring restrictions for files, accelerating activity, providing an eternal home for digital artwork, releasing scientific findings, facilitating data transparency in Web3, and even storing data beyond Earth.

According to the architecture shown in Figure 1, the image obtained from the optical spectrum analyzer is stored in the system. The user transmits images to the backend system for processing. The backend obtains the image and uploads it to IPFS, a decentralized storage system that enables the storage and retrieval of files across a distributed network of nodes. Once the image is posted to the IPFS, it is given a distinct content identifier (CID), which functions as a hash of the image data. This CID serves as an enduring and unchangeable identifier for the image stored on the IPFS. Once the image is successfully uploaded to the IPFS, the backend engages with a smart contract that has been established on the blockchain. The smart contract is tasked with storing the metadata linked to the uploaded image. The backend transmits a transaction to the smart contract, encompassing pertinent information such as the IPFS CID, user information, timestamp, and any more essential metadata. It also verifies the transaction and records the metadata on the blockchain. Subsequently, the transaction that encompasses the image metadata is extracted and incorporated into a block on the blockchain. In this specific instance, Ganache serves as the blockchain network. Ganache [45] is a decentralized application development environment that emulates the functionality of an actual blockchain network. After the transaction is processed and included in a block, the image metadata becomes a permanent and unchangeable part of the blockchain record. During the process of retrieving an image, a user submits a request to the system along with image metadata using a smart contract. The smart contract provides the IPFS CID linked to the image. By utilizing the IPFS CID, the user can retrieve the authentic picture data from the IPFS network. The authenticity of the image can be confirmed by comparing the IPFS CID stored on the blockchain with the CID of the downloaded image.

## 4. Results and Discussion

The proposed methodology is evaluated using two primary criteria: (1) assessment of the FSOs communication system design, and (2) analysis of the blockchain network performance metrics.

### 4.1. Experimental Testbed

In this study, the experimental testbed integrates an FSOs communication system simulated through OptiSystem software Version 7.0, comprising an optical transmitter, channel, receiver, Optical Spectrum Analyzer and BER analyzer components for comprehensive performance analysis. The system is coupled with a blockchain infrastructure utilizing Ganache for the development environment, smart contracts for data management, IPFS for distributed storage, and Web3.py for system integration, enabling secure storage and verification of data.

### 4.2. Evaluate FSOs Design

The results presented in Table 2 and Table 3 demonstrate a comprehensive analysis of the FSOs system performance comparing scenarios with and without EDFAs across various transmission ranges and bit rates. Table 2 shows the analysis for 8 and 12 Gbps, while Table 3 presents data for 16 and 20 Gbps configurations.

From Table 2, at a 10 km transmission distance, the system without an EDFA shows a Q-Factor of 64.5921 at 8 Gbps, which significantly improves to 201.041 with EDFA implementation.

**Figure 2:**
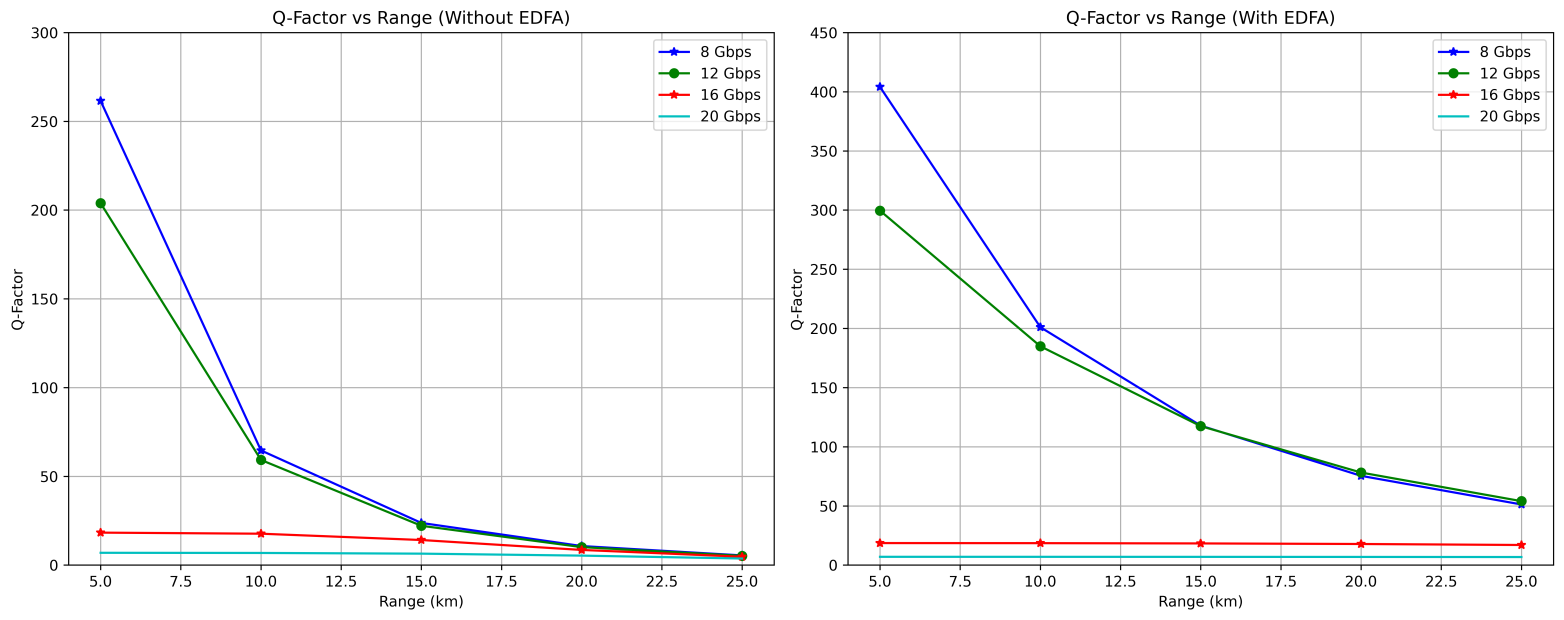
Comparison of Q-Factor values: without using an EDFA (**left**) and with using an EDFA (**right**).

Table 3 reveals that at 15 km and 16 Gbps, the Q-Factor is 14.1135 without an EDFA, improving to 18.2861 with EDFA implementation, as shown in Figure 2. The Bit Error Rate (BER) improvements are notably strong; specifically, Table 2 shows that at 20 km and 12 Gbps, the BER improves from $2.49892\times 10^{-24}$ without an EDFA to 0 with an EDFA, indicating error-free transmission.

As evidenced in both Table 2 and Table 3, the benefits of the EDFA become more pronounced at longer distances. Table 3 shows that at 25 km and 16 Gbps, the system without an EDFA has a BER of $1.41034\times 10^{-6}$, while with an EDFA, it improves to $6.98702\times 10^{-65}$.

The data from both tables illustrate the challenges of increasing bit rates. For example, Table 2 shows that at 25 km, the Q-Factor for 8 Gbps is 5.4449 without EDFA, and decreases to 5.19627 at 12 Gbps.

#### 4.2.1. Analysis of Q Factor

In optical communication networks employing EDFAs, elevated Q-Factor measurements are observed, as demonstrated in Table 2 and Table 3, indicating superior signal integrity and enhanced overall system efficacy. For instance, at 10 km with 8 Gbps, the Q-Factor improves from 64.5921 to 201.041 with EDFA implementation. The presence of elevated Q-Factor values in these EDFA-based systems correlates with improved transmission fidelity, as evidenced by the BER reduction to zero across multiple distances (5–25 km) for both 8 and 12 Gbps configurations.

EDFA is pivotal for reducing signal deterioration, particularly at longer distances. This is clearly demonstrated at 25 km, where the 16 Gbps system’s Q-Factor improves from 4.68169 to 16.9683 with EDFA integration. Conversely, Q-Factor values of FSOs systems without EDFAs show significant deterioration, indicating a decline in signal quality as transmission ranges increase. For example, at 20 Gbps, the Q-Factor decreases from 6.909782 at 5 km to 3.68705 at 25 km without an EDFA, while with an EDFA, it maintains higher values ranging from 6.97469 to 6.78159 across the same distance range.

#### 4.2.2. Analysis of BER

The BER analysis, conducted through OptiSystem’s built-in BER analyzer using pseudo-random bit sequences at various rates (8, 12, 16, and 20 Gbps), shows differences between with-EDFA and without-EDFA FSOs systems. One of the most notable characteristics of FSOs systems using EDFA is their performance, as demonstrated in Table 2, where transmission distances from 15–25 km at both 8 Gbps and 12 Gbps show BER values of 0, indicating that the analyzer could not detect any errors during the simulation period. Conversely, FSOs systems without EDFA exhibit significantly higher BER values with increasing transmission range. As evidenced in Table 3, at 16 Gbps, the BER increases from $6.83964\times 10^{-75}$ at 5 km to $1.41034\times 10^{-6}$ at 25 km. Similarly, at 20 Gbps, the BER deteriorates from $2.42704\times 10^{-12}$ to 0.0011338 over the same distance range, significantly reducing the system’s transmission quality. These specific BER values are obtained from OptiSystem’s BER analyzer and can be verified using the relationship $\mathrm{BER}=0.5\times \mathrm{erfc}(Q/\sqrt{2})$ [46], where *Q* represents the measured Q-factor from this study simulation. While these extremely low BER values show the theoretical capabilities of the system, they would be challenging to verify experimentally due to real-world constraints such as environmental conditions, hardware limitations, and the need for longer data sequences.

### 4.3. Blockchain

To evaluate the blockchain network, three different scenarios are considered: default, optimized, and congested networks. Table 4 presents the parameters used to simulate the network under different techniques. The random delays are represented as base_time + U(0, 1), base_time + U(0, 0.5), and base_time + U(1, 3) for default, optimized, and congested scenarios, respectively.

**Figure 3:**
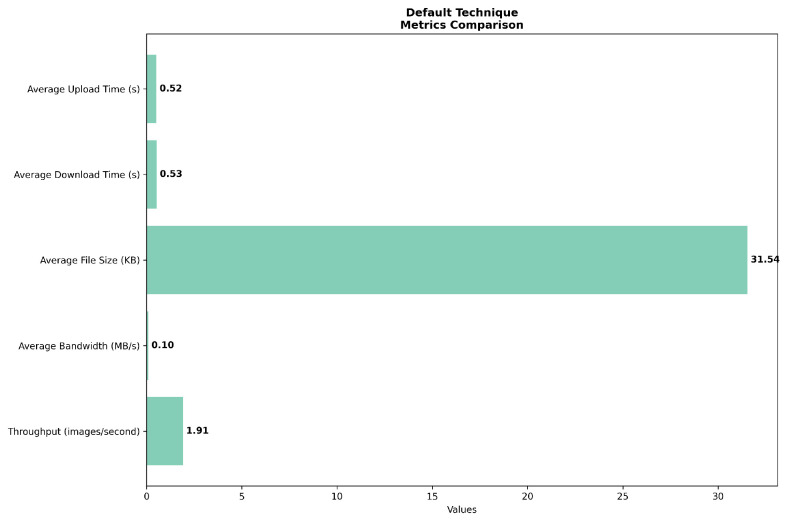
Network simulation on default scenario.

The comparative analysis of system performance across default, congested, and optimized scenarios reveals significant insights into the system’s behavior under varying operational conditions. In the default scenario in Figure 3, the system demonstrates a baseline performance with average upload and download times of 0.52 and 0.53 s, respectively, indicating a balanced handling of bidirectional data transfer. The average bandwidth utilization in this configuration is 0.10 MB/s, with a throughput of 1.91 images per second, establishing a reference point for subsequent comparisons.

**Figure 4:**
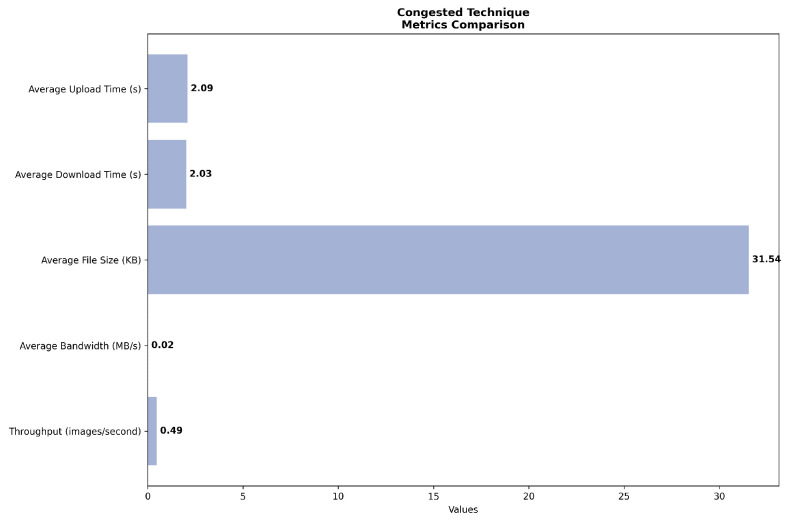
Network simulation on congested scenario.

Under congested conditions in Figure 4, the system exhibits severe performance degradation across all metrics. Upload and download times increase dramatically to 2.09 and 2.03 s, respectively, representing an approximate fourfold increase from the default scenario. This degradation is further evidenced by a significant reduction in average bandwidth to 0.02 MB/s and a concomitant decrease in throughput to 0.49 images per second. These metrics collectively underscore the system’s vulnerability to high-load situations and highlight the critical impact of network congestion on overall system efficiency.

**Figure 5:**
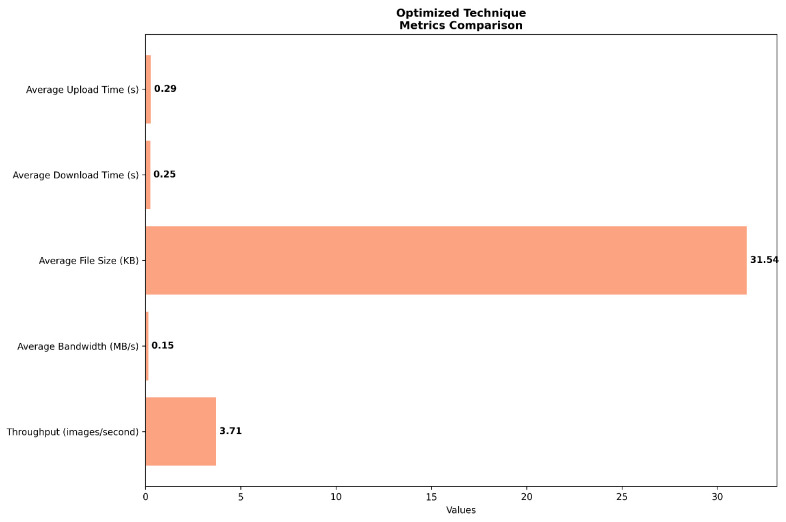
Network simulation on optimized scenario.

The optimized scenario in Figure 5, in contrast, demonstrates marked improvements across all performance indicators. Upload and download times are reduced to 0.29 and 0.25 s, respectively, signifying a substantial enhancement over both the default and congested scenarios. This optimization extends to bandwidth utilization, which increases to 0.15 MB/s, representing a 50% improvement over the default configuration. The most notable enhancement is observed in throughput, which rises to 3.71 images per second, nearly doubling the performance of the default scenario.

It is noteworthy that the average file size remains constant at 31.54 KB across all scenarios, providing a consistent baseline for inter-scenario comparisons. This constancy in file size allows for a more direct attribution of performance variations to the differences in system configurations and network conditions, rather than fluctuations in data volume.

**Figure 6:**
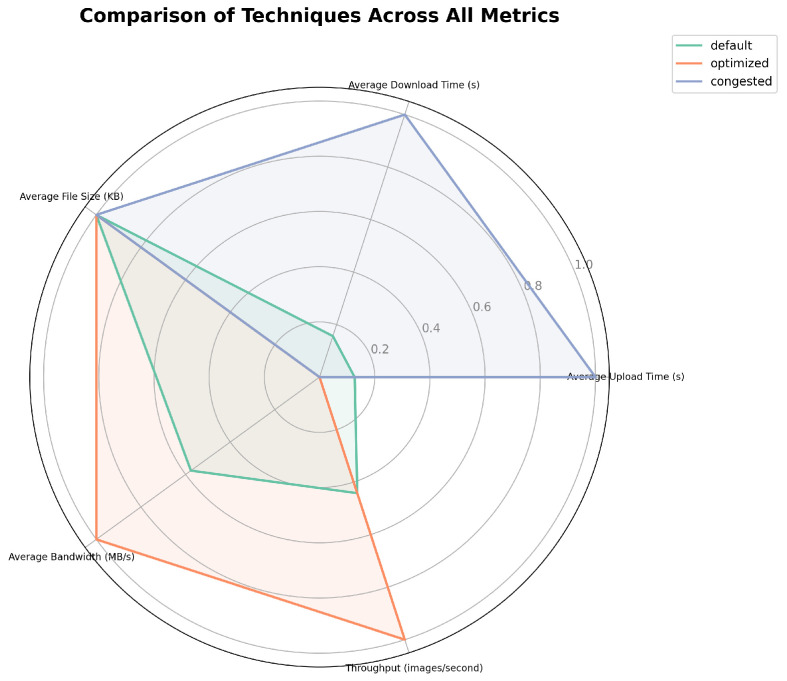
Comparison of network simulation on all scenarios.

Comparing these scenarios reveals several key insights into the system’s behavior and the efficacy of the implemented optimization strategies, as shown in Figure 6. Firstly, the severe performance degradation observed in the congested scenario underscores the critical necessity for robust congestion management mechanisms in maintaining system reliability under high-load conditions. Secondly, the substantial improvements evidenced in the optimized scenario across all metrics—particularly in upload and download times, bandwidth utilization, and throughput—validate the effectiveness of the employed optimization techniques.

Furthermore, the optimization efforts appear to have engendered a more balanced system performance, as evidenced by the convergence of upload and download times in the optimized scenario. This balance suggests a more equitable allocation of system resources and a potential mitigation of the asymmetry observed in the default configuration.

## 5. Applications and Use Cases

This section discusses the proposed method’s use cases and potential impact, which help provide future directions for implementing this study, as discussed in Table 5.

## 6. Conclusions

In the evolving landscape of digital signatures, the integration of blockchain technology with distributed sensor networks represents a significant improvement in tamper-proof data transmission systems. Through the implementation of smart contracts and IPFS, this study provides a robust framework for tamper-proof data validation and storage. The blockchain architecture ensures unprecedented levels of data integrity and transparency while enabling automated verification processes essential for real-time applications. The sensor network implementation showcases the adaptability and efficiency of the system in environmental monitoring and system optimization. Performance metrics from experimental outcomes demonstrate strong capabilities in real-time data processing, establishing a foundation for smart city applications. To facilitate high-speed data transmission within this integrated system, FSOs technology emerges as a complementary solution. The performance analysis encompasses a comprehensive range of operational parameters, examining bit rates of 8, 12, 16, and 20 Gbps across transmission distances from 5 to 25 km, particularly when enhanced with EDFA technology. These extensive testing parameters enable the identification of optimal configurations for various deployment scenarios, ensuring reliable connectivity for the blockchain-validated, sensor-driven network architecture. This synergy between blockchain security, sensor network intelligence, and FSOs transmission presents compelling opportunities for future telecommunications infrastructure.

While the simulation results demonstrate promising performance metrics under controlled conditions (simulation and test network), real-world implementations may experience variations due to environmental factors, network congestion, and atmospheric conditions affecting FSOs transmission quality. Future work may focus on extensive field testing to validate these findings under diverse operational conditions and varying atmospheric turbulence.

## Figures and Tables

**Table 1:** FSOs configuration parameters.

Design Parameters	Values
Laser Power	34.1 dBm
Frequency	1552.52 nm
Attenuation	0.2 db/km
Bit Rate	8 GB/s, 12 GB/s, 16 GB/s, 20 GB/s
Range	5, 10, 15, 20 and 25 km
EDFA Length	10 m

**Table 2:** FSOs system analysis comparison with and without an EDFA (8 and 12 Gbps).

Range	8 Gbps		12 Gbps	
	Q-Factor	BER	Q-Factor	BER
Without EDFA				
5 km	261.522	0	203.885	0
10 km	64.5921	0	59.2321	0
15 km	23.7374	$7.41938\times 10^{-125}$	22.137	$2.27474\times 10^{-109}$
20 km	10.7113	$4.50513\times 10^{-27}$	10.1092	$2.49892\times 10^{-24}$
25 km	5.4449	$2.59161\times 10^{-8}$	5.19627	$1.01019\times 10^{-7}$
With EDFA				
5 km	404.078	0	299.537	0
10 km	201.041	0	184.905	0
15 km	117.834	0	117.422	0
20 km	75.4062	0	78.1821	0
25 km	51.2158	0	54.0116	0

**Table 3:** FSOs system analysis comparison with and without an EDFA (16 and 20 Gbps).

Range	16 Gbps		20 Gbps	
	Q-Factor	BER	Q-Factor	BER
Without EDFA				
5 km	18.2725	$6.83964\times 10^{-75}$	6.909782	$2.42704\times 10^{-12}$
10 km	17.6694	$3.59862\times 10^{-70}$	6.822931	$4.45979\times 10^{-12}$
15 km	14.1135	$1.55927\times 10^{-45}$	6.43107	$6.33487\times 10^{-11}$
20 km	8.52489	$7.58394\times 10^{-18}$	5.32369	$5.08281\times 10^{-8}$
25 km	4.68169	$1.41034\times 10^{-6}$	3.68705	0.00011338
With EDFA				
5 km	18.5811	$2.28094\times 10^{-77}$	6.97469	$1.53263\times 10^{-12}$
10 km	18.5113	$8.36557\times 10^{-77}$	6.96062	$1.69365\times 10^{-12}$
15 km	18.2861	$5.32776\times 10^{-75}$	6.93119	$2.08615\times 10^{-12}$
20 km	17.8006	$3.47995\times 10^{-71}$	6.87587	$3.07932\times 10^{-12}$
25 km	16.9683	$6.98702\times 10^{-65}$	6.78159	$5.93766\times 10^{-12}$

**Table 4:** Network simulation parameters for different techniques.

Parameter	Default	Optimized	Congested
Network Delay	base_time + U(0, 1)	base_time + U(0, 0.5)	base_time + U(1, 3)
Packet Loss	5%	1%	10%
Bandwidth	5 MB/s	10 MB/s	1 MB/s

**Table 5:** Use cases and applications of proposed methodology.

Use Case	Implementation	Research Directions	Potential Impact
Environmental Monitoring [47,48]	Sensor networks using FSOs for data transmission and blockchain for data integrity in environmental monitoring	Resilient FSOs systems for adverse weather Energy-efficient blockchain consensus for sensor nodes Data fusion techniques for multi-parameter sensing	Enhanced climate change studies Improved pollution tracking Better ecosystem management
Smart Cities [49,50]	FSO-enabled high-bandwidth communication between urban sensors with blockchain-based data authentication	Scalable blockchain architectures for city-wide networks Secure multi-hop FSOs routing for urban environments Privacy-preserving techniques for citizen data	Improved urban planning Enhanced energy management Better public service delivery
Healthcare Monitoring [51,52,53]	Wearable health sensors using FSOs for data transmission and blockchain for secure data management	Lightweight cryptography for wearable devices FSO-based body area networks (BANs) Smart contracts for automated health data sharing	Enhanced telemedicine capabilities Personalized healthcare Improved patient data privacy
Agricultural Monitoring [54,55,56]	FSO-enabled precision agriculture sensors with blockchain-based crop data management	Energy-harvesting for long-term sensor deployment FSO-based underground-to-aboveground communication Blockchain marketplaces for agricultural data sharing	Optimized crop yields Reduced resource wastage Improved food security
Industrial Automation [57,58]	FSO-connected industrial sensors with blockchain-based operational data management	FSO-based ultra-reliable low-latency communication Blockchain-based digital twin frameworks Decentralized autonomous organizations for decision-making	Increased production efficiency Enhanced supply chain transparency Improved industrial safety

## Data Availability

Data available upon request.
